# Clodronate as a Therapeutic Strategy against Osteoarthritis

**DOI:** 10.3390/ijms18122696

**Published:** 2017-12-13

**Authors:** Maria Teresa Valenti, Monica Mottes, Alessandro Biotti, Massimiliano Perduca, Arianna Pisani, Michele Bovi, Michela Deiana, Samuele Cheri, Luca Dalle Carbonare

**Affiliations:** 1 Internal Medicine, Section D, Department of Medicine, University of Verona, 37134 Verona, Italy; ale.biotti89@gmail.com (A.B.); michela.deiana@univr.it (M.D.); samuele.cheri@univr.it (S.C.); luca.dallecarbonare@univr.it (L.D.C.); 2 Department of Neurosciences, Biomedicine and Movement Sciences, University of Verona, 37134 Verona, Italy; monica.mottes@univr.it; 3 Biocrystallography Lab, Department of Biotechnology, University of Verona, 37134 Verona, Italy; massimiliano.perduca@univr.it (M.P.); ariannapisani92@gmail.com (A.P.); michele.bovi@univr.it (M.B.)

**Keywords:** clodronate, gene expression, osteoarthritis, progenitor cells, SOX9

## Abstract

Osteoarthritis (OA), the most prevalent musculoskeletal pathology, is mainly characterized by the progressive degradation of articular cartilage due to an imbalance between anabolic and catabolic processes. Consequently, OA has been associated with defects in the chondrocitic differentiation of progenitor stem cells (PSCs). In addition, SOX9 is the transcription factor responsible for PSCs chondrogenic commitment. To evaluate the effects of the non-amino bisphosphonate clodronate in OA patients we investigated *SOX9* gene expression in circulating progenitor cells (CPCs) and in an in vitro OA model. We evaluated pain intensity, mental and physical performance in OA patients, as well as serum biomarkers related to bone metabolism. In addition, in order to improve therapeutic strategies, we assayed nanoparticle-embedded clodronate (NPs-clo) in an in vitro model of chondrogenic differentiation. Our data showed upregulation of *SOX9* gene expression upon treatment, suggesting an increase in chondrocytic commitment. Clodronate also reduced osteoarticular pain and improved mental and physical performance in patients. Furthermore, NPs-clo stimulated *SOX9* expression more efficaciously than clodronate alone. Clodronate may therefore be considered a good therapeutic tool against OA; its formulation in nanoparticles may represent a promising challenge to counteract cartilage degeneration.

## 1. Introduction

Osteoarthritis is a very common condition, covering 80% of all rheumatic disease, and being the main cause of population morbidity in the elderly [1]. Its prevalence increases with age, affecting especially females [2]. Its pathogenesis is related to environmental and genetic factors, some of which are still unknown [3]. Recently, osteoarthritis has been identified as a condition affecting the entire joint, not only the cartilage district [4]. Moreover, subchondral bone alterations, included osteoporosis areas, are connected to cartilage damage and caused by pro inflammatory cytokines (such as TNFα, IL6 and IL1β) and metalloproteinase (MMP) release by macrophages. Pro inflammatory molecules can bind receptors on chondrocytes surface and alter their metabolism, but they can also reduce mesenchymal stem cells (MSCs) chondrogenic differentiation [5]. All these aspects alter cartilage regeneration and subchondral bone metabolism, leading to high remodelling areas, sclerosis, microfractures, cysts and periarticular osteophytes [6,7]. Considering that both osteoarthritis and osteoporosis share these pathological changes, we tested the efficacy of clodronate as a new promising drug capable of modifying OA (osteoarthritis) natural history.

Bisphosphonates are synthetic, non-hydrolyzable analogs of pyrophosphate that contain a P-C-P core and two side chains, named R1 and R2, bound to the central carbon. According to R2 chain characteristics, they can be distinguished into two major groups: nitrogen (N-BF) and non-nitrogen (NN-BF) bisphosphonates [8,9]. Both categories inhibit osteoclast’s bone resorptive action, although in different ways. N-BFs mode of action has been identified recently. Once incorporated into cells, these compounds inhibit farnesyl diphosphonate synthase (FPPS) in osteoclasts, thereby preventing the formation of isoprenoid lipids required for the prenylation of small GTPases, such as Rac, Rho and Ras. The loss of prenylated proteins accounts for osteoclastic drawbacks regarding citoskeletal rearrangement and ruffled border formation. N-BFs show a higher antiresorptive efficacy compared to NN-BFs; however, they also have a pro-inflammatory activity ascribable to isopentyl pyrophosphate backlog in Tγδ limphocytes citosol, due to FPPS inhibition [10,11,12,13]. This may be the cause of flu-like symptoms, observed especially upon intravenous administration [14]. Conversely, NN-BFs show anti-inflammatory effects due to their inhibition of macrophagic release of NO and pro- inflammatory mediators and their proapoptotic action [15,16,17]. For these reasons, NN-BFs have been tested for their ability to reduce inflammatory osteoarthritis and also, in animal models, to prevent acute phase reaction after N-BFs injection [18,19,20,21]. Clodronate (dichloromethylene-1,1-bisphosphonate) is a halogenated NN-BF, with proven antiresorptive efficacy in a variety of diseases associated with excessive bone resorption, including hypercalcemia of malignancy, osteolytic bone metastases, primary hyperparathyroidism and Paget’s disease. As for other bisphosphonates, its affinity for bone matrix is not relevant, so patients need a long-term therapy with short intervals between doses, in order to obtain clinical benefits. Recently, clodronate efficacy has been tested upon intra-articular (erosive osteoarthritis) and intra-dermic administration [20]. Furthermore, its anti-inflammatory and analgesic efficacy, possibly related to its pro-apoptotic action on macrophages, may be beneficial for chondrogenesis. In fact, release of NO and pro-inflammatory cytokines (such as TNFα and IL1β) [15,16,17] may promote cartilage erosion, subchondral bone alterations, and inhibit progenitors’ maturation into chondrocytes in OA early stages. Clodronate could therefore stimulate cellular differentiation by regulating inflammatory pathways [22].

MSCs feature promising sources for cell-based therapeutic strategies. They are generally defined as self-renewable, multipotent progenitor cells with the ability to differentiate into several mesenchymal lineages, including bone, cartilage, adipose and muscle tissues. SOX9 is the master transcription factor for MSC differentiation into chondrocytes, exerting its role along the whole pathway [22]. SOX9 expression is regulated by BMPs and it activates many extracellular matrix (ECM) genes such as *COL2A1*, *COL9A1*, *COL11A2* and *ACAN* (aggrecan) [23,24,25].

## 2. Results

### 2.1. Patients

Average age, height, weight, BMI and menopause age were 71.8 ± 7 years, 153 ± 5.8 cm, 64.2 ± 8.6 kg, 27.4 ± 3.5 kg/m^2^ and 46.5 ± 7 years, respectively. Among bone metabolism parameters, only CTX values showed a significant reduction at the end of the study (0.25 ± 0.08 ng/mL at M6 vs. 0.39 ± 0.19 ng/mL at M0; *p* < 0.05). Moreover, 25 hydroxyvitamin D levels did not manifest relevant variations during therapy, and they maintained average values over insufficiency cut-off (20 ng/mL) (Table 1).

Moreover, visual analogue pain scale (VAS) showed relevantly decreased scores at the end of treatment in older women. Numerical rating pain scale (NRS) showed a significant decrease of symptoms after three months in the same group (*p* < 0.05 for both). A decrease in pain intensity likely warranted patients a better quality of life. This point is confirmed by significant increases of ISM and ISF scores at the end of treatment, compared to basal values. VAS and NRS average pain scores decreased rapidly; however, only NRS pain score reached a significant improvement after drug assumption (4.27 ± 2.06 at M6 vs. 6.00 ± 2.34 at M0; *p* = 0.01). ISF and ISM indexes of SF36 survey both reached relevant improvement after 6 months (ISF score: 43.04 ± 6.73 at M6 vs. 36.89 ± 12.21 at M0; *p* < 0.05; ISM score: 45.75 ± 3.86 at M6 vs. 42.54 ± 4.87 at M0; *p* < 0.05) (Table 2).

### 2.2. Gene Expression in OA Patients’ CPCs

**Figure 1:**
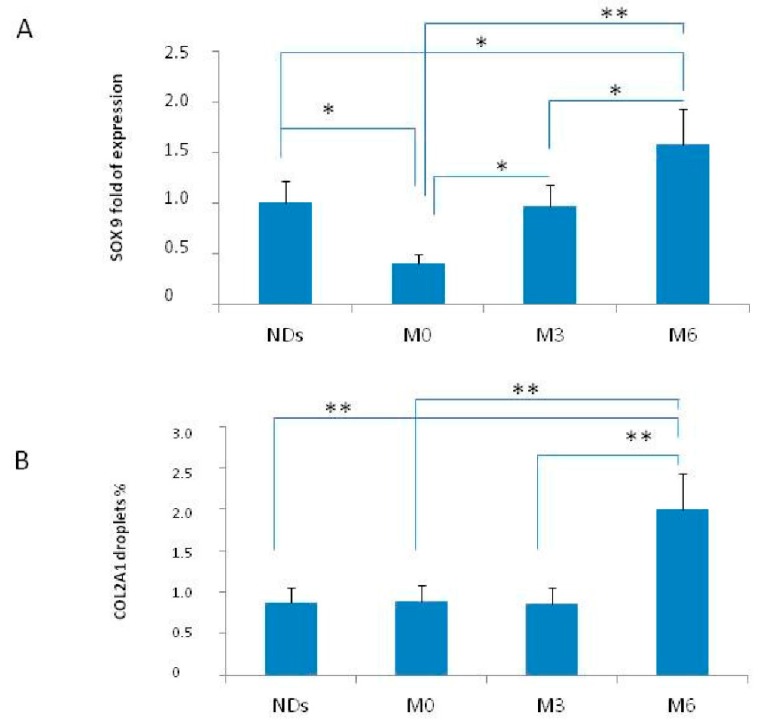
*SOX9* (**A**) and *COL2A1* (**B**) fold of expression in CPCs of Normal Donors (NDs) and patients at baseline (M0), after 3 (M3) and 6 (M6) months. * *p* < 0.05; ** *p* < 0.001.

CPC cluster differentiation (CD) expression patterns were similar in normal donors (NDs) and Patients (Table 3). Therefore, SOX9 expression was analyzed in CPCs from patients and NDs at M0, M3 and M6, respectively. Gene expression levels were monitored in all samples. *SOX9* average expression in patients increased constantly during the study, matching (M3) and then exceeding (M6) control levels (Figure 1A). Interestingly, COL2A1 expression in patients also increased during the study (Figure 1B).

### 2.3. Chitosan-Hyaluronic Acid-Clodronate Embedded Nanoparticles

Synthesized clodronate nanoparticles were analyzed by dynamic light scattering and showed a single peak at 135.4 nm with a polydispersity index (pdI) of 0.922 and a surface charge of 25.5 mV.

The encapsulation efficiency (EE%) was estimated to be 64.9%. It was calculated from a clodronate calibration curve. These nanoparticles were used for all further experiments.

### 2.4. Gene Expression in the In Vitro OA Model 

**Figure 2:**
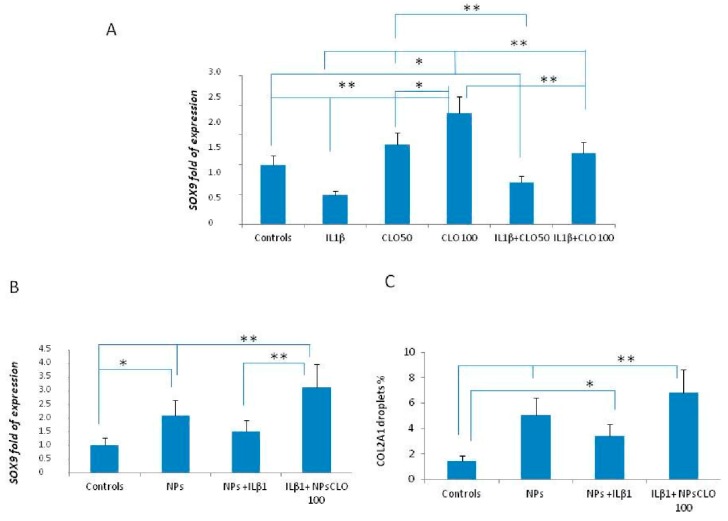
Effects of clodronate in mesenchymal stem cells (MSCs). *SOX9* fold of expression in MSCs treated with and w/o clodronate in chondrogenic medium in the presence or absence of ILβ1 (**A**). *SOX9* (**B**) and *COL2A1* (**C**) fold of expression in chitosan and hyaluronic acid empty nano particles (NPs) or clodronate embedded nanoparticles in chindrogenic medium with or w/o ILβ1. The synergistic action of NPs and clodronate is noteworthy. * *p* < 0.05; ** *p* < 0.01.

**Figure 3:**
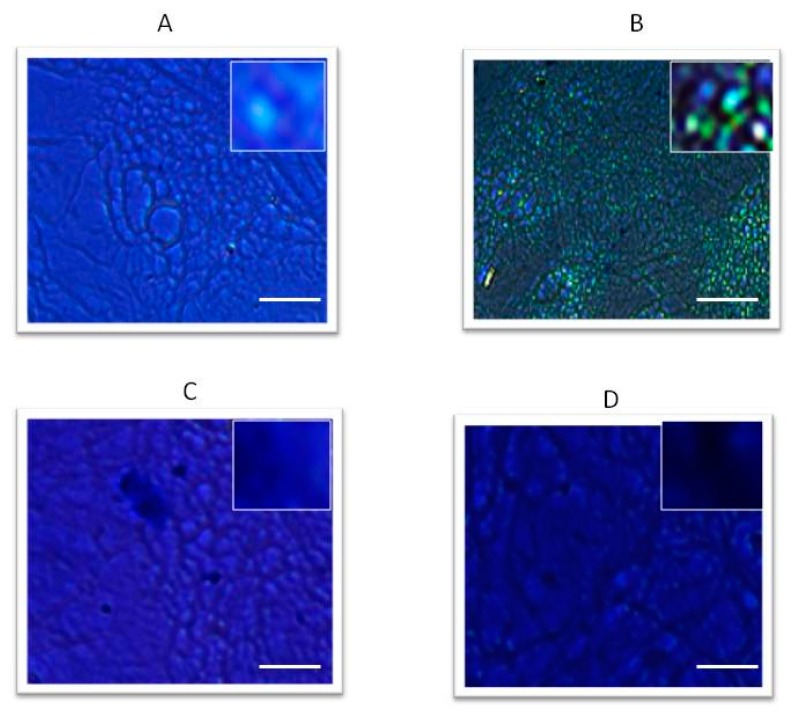
Alcian blue staining. After 21 days of culture, cells were fixed and stained with alcian blue in order to evaluate GAGs production. Control (**A**), cells treated with GFP nanoparticles alone (**B**); in the presence of ILβ1 (**C**); clodronate embedded NPs in the presence of ILβ1 (**D**). Scale bar 150 μm; insert 80×.

As we did in vivo, we studied clodronate effects in vitro, in cultured MSCs. *SOX9* expression was surveyed in order to evaluate chondrogenic differentiation. All results were reported as normalized values compared to their expression at the end of the differentiation process (in specific mediums) without IL1β and/or clodronate addition to cultures. The in vitro experiments confirmed IL1β inhibition of chondrogenic maturation. This pro-inflammatory cytokine halved MSCs ability to differentiate. On the other hand, clodronate increased MSCs’ potential to undergo chondrogenic differentiation in a dose dependent way. We then added two different combinations of IL1β + clodronate (50 nM and 100 nM) to the cultures. At the lower dose, the drug inhibited cytokine pro-inflammatory action only partially; but at the higher dose, clodronate action exceeded IL1β inhibition, stimulating MSCs maturation (Figure 2A). In order to improve the therapeutic effect against OA, we tested customized nanoparticles produced with molecules which are employed in cartilage tissue engineering, as chitosan and hyaluronic acid [26]. Nanoparticles embedded-clodronate exhibited a stronger effect in counteracting IL1β inhibition of *SOX9* (Figure 2B) and *COL2A1* (Figure 2C) expression. Notably, MSCs cultured with chondrogenic differentiation medium in the presence of clodronate alone or embedded in nanoparticles, exhibited a strong positive staining with alcian-blue indicating the production of glycosaminoglycan (GAG) and therefore the chondrogenic maturation (Figure 3).

## 3. Discussion

Recent hypotheses regarding the pathogenesis of osteoarthritis have confirmed that subchondral bone alterations, including osteoporosis areas, appear at an early stage of the disease, influence its evolution, and are also associated to cartilage damage [1]. Moreover, these signs also occur in other bone diseases characterized by an excessive bone resorption. Pro-inflammatory cytokines, such as TNFα and IL1β, aggravate cartilage erosion due to the secretion of MMPs and other factors which destroy articular tissues and to the inhibition of progenitor cells differentiation into mature chondrocytes [27]. Articular pain depends on sinovitis which is related to macrophage inflammatory activity and bone marrow lesions, consequent to excessive bone resorption [15]. Clodronate (as other NN-BFs), with its antiresorptive and anti-inflammatory action, appears therefore an ideal candidate for osteoarthritis therapy, possibly capable of influencing the natural history of this disease. Clodronate also exerts, through its interaction with purinergic receptors on chondrocyte surface, an anabolic function on this cellular type, enhancing ECM components secretion [28].

Thus, in our study, we evaluated for the first time the in vivo and in vitro effects of clodronate on peripheral blood MSCs differentiation. We also evaluated its influence on bone metabolism, osteoarticular pain, mental and physical performance.

Our outcomes demonstrated, for the first time, that intramuscular 200 mg clodronate weekly assumption stimulates in vivo MSCs maturation toward the chondrogenic lineage. Clodronate strongly increased SOX9 expression after three and six months treatment, compared to patients’ basal value. In addition, after six months of treatment, patients’ *SOX9* and *COL2A1* expression exceeded NDs’. Transcription factor *SOX9* induces mesenchymal cells differentiation into chondrocytes, upregulating specific chondrogenic genes such as *COL2A1* [29]. Clodronate also exerts analgesic effects. NRS pain scale showed a significant decrease at the end of treatment in both groups. Bivariate correlations also evidenced direct concordance between mean ISF scores and mean 25 hydroxyvitamin D levels, suggesting that the hormone influences physical performance. Several studies have demonstrated that higher hormone levels enhance physical performance and strength in the elderly [30].

Clodronate, as other bisphosphonates, inhibits bone resorption since the first months of assumption: this is confirmed by the significant decrease in CTX values after six months, compared to basal values. We also recall that 25 hydroxyvitamin D mean levels did not change noticeably during our study, and constantly remained above the insufficiency cut-off (20 ng/mL): we can therefore state that SF36, VAS and NRS outcomes are not influenced by the hormone blood levels. Our results also confirmed that short-term clodronate therapy does not affect renal function: serum creatinin did not increase significantly after six months of drug assumption.

In order to analyze the molecular effects of clodronate in an OA in vitro model, we cultured a human MSC line with IL1β, an inflammatory cytokine involved in OA pathogenesis [31]. Interestingly, our data confirmed the chondrogenic differentiation induced by clodronate observed in CPCs obtained from treated patients. In fact, *SOX9* gene expression increased significantly in a dose-dependent manner in cells treated with clodronate. This effect was observed even in co-occurrence with IL1β. Clodronate, alone or embedded in nanoparticles, was able to stimulate the condrogenic maturation, proven by the alcian blue staining data.

These outcomes strengthen the idea that clodronate stimulates chondrogenic differentiation of precursors and may hinder effectively the pathogenesis and progression mechanisms of OA.

In addition, our finding that clodronate embedded in NPs may increase further SOX9 expression stimulates the search for new therapeutical strategies against osteoarthritis. Due to its multiple mechanisms of action over all the different pathways involved in OA pathogenesis, clodronate appears an ideal candidate for new therapies against this condition. However, additional studies are necessary in order to verify whether clodronate is able to influence osteoarthritis natural history.

## 4. Patients, Materials and Methods

### 4.1. Subjects

Written informed consent was obtained from all participants and the study was approved by the Ethical Committee of Azienda Ospedaliera Universitaria Integrata of Verona, Italy (number 1538, 3 December 2012).

We selected 23 female patients, (age: 60–83 years), recruited through the Veneto’s Specialistic Regional Center for Skeletal and Degenerative Diseases. Patients were treated with clodronate I.M. 200 mg weekly. All subjects were affected by spondiloarthritis evaluated by dorso-lumbar X-rays. All patients at entry were administered Dibase 100,000 UI, once a month.

Exclusion criteria were: any cause of secondary osteoporosis, antiresorptive therapy (e.g., bisphosphonates, strontium ranelate, denosumab), in the previous 12 months, bone metabolism modifying drugs, (e.g., statins or tiazidics), vitamin D insufficiency (<20 ng/mL), NSAIDs, hormonal replacement therapy, smoking, alcoholism, vertebral fractures (defined with Genant criterias at the morphometric evaluation of spine).

The Control group consisted of 5 healthy females (age: 25–30 years, height 154 ± 3.2 cm, weight 63.4 ± 4.2 kg, BMI 26.7 kg/m^2^). All subjects were in the bone mass peak age. Exclusion criterias were: any cause of primary or secondary osteoporosis and osteoarthritis.

Three blood samples were obtained by venipuncture from each patient at three different time points named M0 (before treatment), M3 and M6 (after 3 and 6 months, respectively, of treatment). circulating progenitor cells (CPCs) were isolated from each blood sample. At the same time, VAS, NRS and SF36 surveys were completed by each participant. Two additional blood samples were obtained from each subject at the beginning (M0) and at the end of the study (M6) for bone metabolism parameters evaluation. We quantified serum blood calcium, PTH, 25 hydroxyvitamin D, CTX (C-terminal peptide of collagen type I) serum creatinin and urinary calcium excretion rate levels in order to exclude secondary osteoporosis causes and to evaluate therapy influences on their expression at the end of study. M0 and M6 average scores were calculated for each bone metabolism index. Outcomes are expressed as mean ± standard deviation.

Control group subjects, upon written consent, were submitted to a single venipuncture in basal conditions (M0) for the isolation of CPCs. Results were calculated both for the entire study population and for patients aged ≥70 alone (6 subjects). We considered this subgroup as electively representative of OA affected people, since the disease incidence peak falls after age 70. 

### 4.2. VAS, NRS, SF36 Surveys

Each patient completed anonymously the three surveys at each time point (M0, M3, M6). VAS and NRS surveys evidenced osteoarticular pain scores: in VAS, we asked patients to position a cross sign-according to the gravity of their symptoms, within a line spanning from no pain to high intensity pain. In NRS, patients were asked to associate a number to their pain, in a 0–10 points scale. SF36 survey instead consisted of a list of questions about life quality. We obtained also scores about mental and physical performance (ISM and ISF, respectively). At the end of treatment, we calculated M0, M3 and M6 average scores for VAS and NRS pain scales, ISM and ISF indexes. All results were expressed as mean ± standard deviation.

### 4.3. Circulating Progenitor Cells (CPCs)

CPCs were isolated from 50 mL of heparinized blood using two Ficoll procedures to deplete hematopoietic cells by antibodies cocktail, as previously reported [32,33]. The enriched cells obtained were washed in phosphate-buffered saline (PBS) and phenotype analysis was performed as previously described [33]. Then, CPCs were analyzed for gene expression.

### 4.4. Chondrogenic Differentiation of Mesenchymal Stem Cells

We used hMSCs (PromoCell) to analyze the effects of clodronate, alone or embedded in nanoparticles, on chondrogenic differentiation. We chose commercial MSCs in order to avoid confounding effects of different circulating growth factors as well as cytokines. Cells were plated at a density of 5 × 10^4^ cells per well into 48-well plates in chondrogenic differentiation medium (DMEM, with 100 nM dexamethasone, 200 umol ascorbic acid and 10 ng/mL TGF β), for 21 days at 37 °C in humidified atmosphere with 5% CO_2_. Medium was changed every 2 days.

### 4.5. Nanoparticles Synthesis

Bisphosphonate nanoparticles were prepared using chitosan and hyaluronic acid applying the ionotropic gelation method. 600 μg of clodronate were dissolved in 0.6 ml of distilled water and added to 100 mL of chitosan solution (100 μg/mL in acetic acid 1% pH 5) under magnetic stirring for 20 min. 30 mL of hyaluronic acid solution (115.2 μg/mL in 100 mM acetic acid pH 5) were added dropwise to the emulsion under stirring for 1 hour to enable complete stabilization of the system.

Green fluorescent protein (GFP) embedded nanoparticles, were prepared with the same protocol substituting clodronate with 1 mg of GFP. Finally, all nanoparticles (NPs) were divided into aliquots and lyophilized.

The nanoparticles mean size and zeta potential were estimated using the dynamic light scattering (DLS) technique (Nano ZetaSizer ZS, ZEN3600, Malvern Instruments, Malvern, Worcestershire, UK), re-suspending the synthesized NPs in PBS buffer (137 mM NaCl, 2.7 mM KCl, 10 mM Na_2_HPO_4_ and 1.8 mM KH_2_PO_4_) at the final concentration of 5 mg/mL with the sample cell temperature fixed at 25 °C.

The encapsulation efficiency was calculated from a clodronate UV absorbance calibration curve prepared with different amounts of the drug dissolved in an aqueous solution containing 1.5 mM CuSO_4_ and 1.5 mM HNO_3_ at pH 3. Absorbance was recorded at 261 nm using empty nanoparticles absorbance as basic correction [34].

### 4.6. In Vitro Treatments

Six different combinations of supplements were added to the cell cultures during chondrogenic differentiation. In detail: IL1β alone, NPs alone, Clodronate 50 nM, NPs + IL1β, Clodronate 100 nM, IL1β + Clodronate 50 nM, IL1β + Clodronate 100 nM, IL1β + Clodronate-embedded nanoparticles 100 nM. Noteworthy, IL1β, an inflammatory cytokine, was added in order to mimic OA conditions as previously reported [31]. Cultures without supplements were taken as controls. Three independent experiments were performed for each condition.

### 4.7. Total RNA Extraction

Total RNA was extracted from each cell pellet using the RNA assay Minikit (Quiagen, Hilden, Germany) with DNAse I treatment. The amount of extracted RNA was quantified by measuring the absorbance at 260 nm. The purity of RNA was checked by measuring the ratio of the absorbance at 260 and 280 nm, where a ratio ranging from 1.8 to 2.0 was taken to be pure.

### 4.8. Reverse Transcription

First-strand cDNA was generated, according to the manufacturer’s protocol, using the First Strand cDNA Synthesis Kit (GE Healthcare, Little Chalfont, UK), with random hexamers, reverse transcriptase and 4 dNTPs. 1 μg RNA was employed in each reaction.

### 4.9. Real Time RT-PCR

PCR was performed in a total volume of 50 µL containing 1× Taqman Universal PCR Master Mix, no AmpErase UNG and 5 µL of cDNA from each sample; pre-designed *SOX9*-specific primers and probe set was obtained from Assay-on-Demand Gene Expression (Thermofisher Corporation, Waltham, MA, USA). Real Time RT-PCR reactions were carried out in multiplex. The real-time amplifications included 10 min at 95 °C, followed by 40 cycles at 95 °C for 15 s and at 60 °C for 1 min. Thermocycling and signal detection were performed with ABI Prism 7300 Sequence Detector. Signals were detected according to the manufacturer’s instructions. *SOX9* gene expression levels during chondrogenic differentiation were calculated in triplicate for each sample after normalization against the housekeeping genes (β_2_ microglobulin and GADPH), using the relative fold expression differences. Average *C*_t_ value was used to calculate the relative mRNA expression levels of the PCR targets, using the comparative *C*_t_ method with the equation: relative expression = 2^−[*C*t (target) − *C*t (reference gene)]^ × 100.

### 4.10. Ct DATA

*C*_t_ values for each reaction were determined using TaqMan SDS analysis software. For each amount of RNA tested triplicate *C*_t_ values were averaged. Since *C*_t_ values vary linearly with the logarithm of the amount of RNA, this average represents a geometric mean.

### 4.11. ddPCR

In order to analyze the expression of COL2A1, which is scarcely expressed in CPCs, we performed the digital droplet PCR (ddPCR). 5 μL of RNA samples (0.2 ng/μL) were added to 10 μL of ddPCR supermix for no UTP probes, and to 1 μL of COL2A1 TaqMan probe (Applied Biosystems). The mix was applied to QX200 droplet generator (BioRad, Hercules, CA, USA) with 70 μL of oil. Droplets were transferred into a 96 well plate and heat-sealed with tinfoil sheet. Thermocycling conditions were as follows: pre-incubation at 95 °C for 10 min, amplification at 95 °C for 30 s, annealing at 60 °C for 1 min, for 40 cycles, heat inactivation at 98 °C for 10 min. Plates containing droplets were placed in a QX200 droplet reader, which analyses droplets individually, through a two color detection system (FAM and VIC). Results were processed by QuantaSoft (BioRad) according to the manufacturer’s instructions.

### 4.12. Alcian Blue Staining

Alcian blue staining was performed as previously reported [35]. Briefly, after 21 days of culture, the cell slides were fixed with 95% methanol and then stained with 1% Alcian blue 8GX HCl overnight. Subsequently, cell slides were gently washed and observed under microscope.

### 4.13. Statistic Analysis

Statistical analyses were performed using SPSS 21.0 for Windows operative system. For multiple comparisons, statistical analysis was assessed by one-way ANOVA. Results were expressed as mean ± standard deviation.

## 5. Conclusions

We conclude that clodronate assumption, over a six-month period, stimulated significantly in vivo MSCs differentiation toward the chondrogenic lineage. This drug also reduced osteoarticular pain, improved mental and physical performance and, according to other studies, also diminished bone resorption after the first months of assumption. Finally, clodronate stimulated, in a dose dependent manner, chondrogenic differentiation of MSCs also in vitro and we demonstrated that it can counteract the inflammatory inhibition of chondrogenic differentiation.

## Figures and Tables

**Table 1:** Biochemical data.

Parameters	Basal	Treatment	*p*	Normal Range
Serum Calcium (mg/dL)	9.28 ± 0.33	9.60 ± 0.40	NS	8.41–10.42 mg/dL
PTH (pg/mL)	42.00 ± 19.20	41.23 ± 21.38	NS	10–65 pg/mL
Vit D (ng/mL)	31.45 ± 14.40	38.89 ± 9.31	NS	<30 ng/mL insufficiency <20 ng/mL depletion
CTX (ng/mL)	0.39 ± 0.19	0.25 ± 0.08	*p* < 0.05 vs. CTX M0	0.1–0.7 ng/mL
Creatinin (mg/dL)	0.74 ± 0.12	0.77 ± 0.13	NS	0.49–1.19 mg/dL
Urinary Calcium Excretion Rate (mmol/mmol creatinine)	0.42 ± 0.23	0.42 ± 0.22	NS	<0.57 mmol/mmol creatinin

NS: not significant; PTH: parathyroid hormone; Vit D: vitamin D; CTX: C-terminal telopeptide.

**Table 2:** VAS (visual analogue pain scale) and NRS (numerical rating pain scale pain scales) during the study.

VAS M0	VAS M3	VAS M6	NRS M0	NRS M3	NRS M6
5.30 (±2.7)	4.2 (±2.1) *p* < 0.05 vs. VAS M0	3.9 (±2.2) *p* < 0.01 vs. VAS M0	5.7 (±2.2)	4.9 (±2.0) *p* = NS	4.9 (±2.2) *p* < 0.01 vs. NRS M0
ISF MO	ISF M3	ISF M6	ISM M0	ISM M3	ISM M6
36.8 (±12.9)	39.6 (±8.9) *p* = NS	428 (±6.5) *p* < 0.01 vs. ISF M0	43.2 (±4.7)	45.2 (±6.4) *p* = NS	45.6 (±37) *p* < 0.05 vs. ISM M0

**Table 3:** Cell phenotype of CPCs (Circulating Progenitor Cells) after depletion.

Cluster Differentiation	NDs	M0	M3	M6
CD3	Undetectable level	Undetectable level	Undetectable level	Undetectable level
CD14	0.34 ± 0.05%	0.4% (±0.02)	0.34% (±0.4)	0.37% (±0.05)
CD19	Undetectable level	Undetectable level	Undetectable level	Undetectable level
CD45	2.35 ± 0.37%	1.51% (±0.6)	2.16% (±0.3)	1.6% (± 0.8)
CD34	Undetectable level	Undetectable level	Undetectable level	Undetectable level
